# High-temperature superconductivity as viewed from the maximum hardness principle

**DOI:** 10.1007/s00894-018-3777-6

**Published:** 2018-08-14

**Authors:** Wojciech Grochala, Mariana Derzsi

**Affiliations:** 1 Center of New Technologies, University of Warsaw, Żwirki i Wigury 93, 02089 Warsaw, Poland; 2 Advanced Technologies Research Institute, Faculty of Materials Science and Technology in Trnava, Slovak University of Technology in Bratislava, J. Bottu 25, 917 24 Trnava, Slovak Republic

**Keywords:** Metal, Superconductor, Density of states, Hardness, Critical superconducting temperature, Band gap

## Abstract

The Maximum Hardness Principle – and its reformulation by Chattaraj as the Minimum Polarizability Principle – is an immensely useful concept which works in support of a chemical intuition. As we show here, it may also be used to rationalize the scarcity of high-temperature superconductors, which stems – inter alia – from rarity of high-density of state metals in Nature. It is suggested that the high-temperature oxocuprate superconductors as well as their iron analogues – are energetically metastable at T ➔ 0 K and p ➔ 0 atm conditions, and their tendency for disproportionation is hindered only by the substantial rigidity of the crystal lattice, while the phase separation and/or superstructure formation is frequently observed in these systems. This hypothesis is corroborated by hybrid density functional theory theoretical calculations for Na- (thus: hole) or La- (thus: electron) doped CaCu(II)O_2_ precursor. Non-equilibrium synthetic methods are suggested to be necessary for fabrication of high-temperature superconductors of any sort.

**Graphical abstract:**
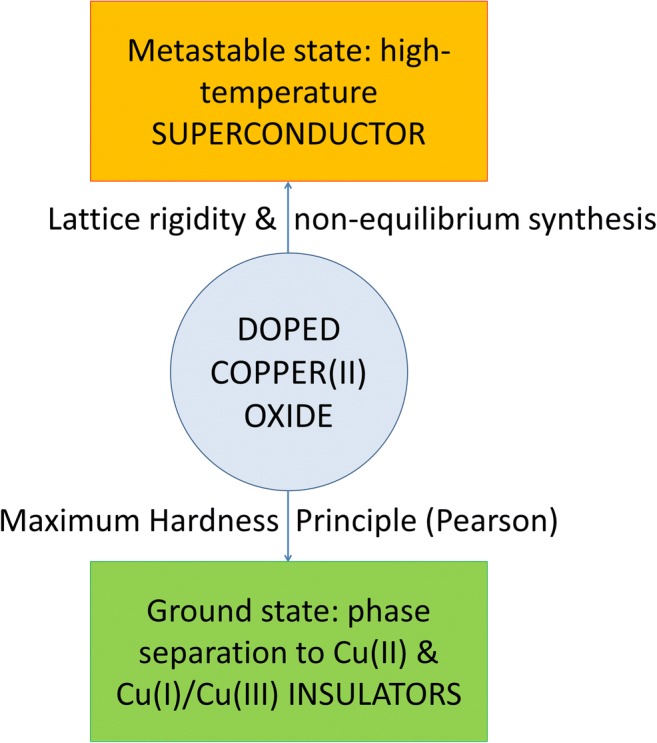
Doped oxocuprate superconductors are shown to be unstable with respect to phase separation (disproportionation) in accordance with the Maximum Hardness Principle; their metastability is mostly due to rigidity of [CuO_2_] sheets and preparation using high-temperature conditions

Doped oxocuprate superconductors are shown to be unstable with respect to phase separation (disproportionation) in accordance with the Maximum Hardness Principle; their metastability is mostly due to rigidity of [CuO_2_] sheets and preparation using high-temperature conditions

## The dream of room-temperature superconductivity

Superconductivity is a fascinating collective quantum phenomenon which consists of unhindered transport of electric current (supercurrent) through a solid [1]. Superconductivity finds many uses, including construction of powerful magnets, fast processors, and Superconducting Magnetic Energy Storage devices, but its most important economic aspect is that it helps to save vast amount of money (or fossil energy resources) if used for transfer of large densities of electric current. To date, superconducting cables have been successfully applied when placed between the power plant and the energy distributing centers at the city outskirts, as in Detroit [2] or Essen; mega amperes of electric current density may be safely transmitted using this technology (http://www.aist.go.jp/aist_e/list/latest_research/2018/20180305/en20180305.html). The widespread use of the superconductor technology is, however, limited by the relatively low superconducting critical temperature, T_C_, values, for most types of superconductors known; this prevents superconducting cables to be used at ambient temperature conditions. The current T_C_ record is 138 K (at 1 atm; for oxocuprate material [3]) and 203 K (at impractically high pressure exceeding 2 mln atm; for sulfur-hydrogen system [4]). As comfortable illusions are unfold of the energy- and cost-saving world, the quest for the room-temperature superconductor continues and keeps attracting many new acolytes of science.

Regretfully, as one may readily notice, there is currently just one family of high-T_C_ superconductors at 1 atm conditions, i.e. oxocuprates, the “high-T_C_ “label being traditionally defined as T_C_ > T_boil_(N_2_) [5]. As the world is filled with zillions of low-T_C_ materials, encompassing virtually any chemical class of alloys and chemical compounds (including organic ones), this scarcity of high-T_C_ materials is striking and disturbing. Indeed, collective quantum phenomena such as superfluidity or superconductivity may too easily be destroyed by thermal excitations. While hope never dies and many researchers show considerable optimism (https://www.houstonchronicle.com/local/history/medical-science/article/Houston-scientist-Dr-Paul-Chu-upends-the-physics-8406495.php), are there any rational reasons why the situation might be considerably improved in the near future?

In this contribution we try to understand the scarcity of high-T_C_ - and even moderate-T_C_ materials (77 K > T_C_ > 23 K for Nb_3_Ge) - by applying the reasoning based on the Maximum Hardness Principle (MHP) from Pearson [6].

## The maximum hardness principle and its application to metallic state

The Maximum Hardness Principle [6] – and its reformulation by Chattaraj as the Minimum Polarizability Principle [7] – is an immensely useful qualitative concept which works in support of a chemical intuition, and it may be applied to a vast array of important problems [8, 9]. The hardness in question, η, is the *electronic* (and not mechanical) hardness, i.e. a measure of the ease of deformation of the electronic density. Within the conceptual density functional theory (DFT) framework, η is the second derivative of system’s energy with respect to the number of electrons.

The MHP in its original formulation states that the chemical system adopts such geometry of nuclei that the associated electronic hardness is maximized. This statement, first voiced by Pearson [6], founder of the hardness concept [10], in fact is a *generalized* form of MHP, abbreviated here as GMHP [8, 9]. The GMHP does not universally hold and it cannot be proved, since it has one important exception – namely it does not apply to totally symmetric vibrations of the chemical system. In other words, if a chemical system with a given nuclear geometry (corresponding to equilibrium structure) is compressed (squeezed) along the totally symmetric vibrational coordinate, the hardness will inevitably increase. Consequently, GMHP should be understood in more practical sense in such a way that among all nuclear positions, which a chemical system may freely adopt without any external constraints, the one which corresponds to a ground state usually exhibits the largest electronic hardness; other less stable nuclear configurations (which may correspond to metastable forms, i.e. isomers or polymorphs in the solid state) tend to have smaller electronic hardness (cf. Ref. [8, 9] and numerous examples from literature discussed therein). This behaviour is nicely exemplified by elemental tin for which the semimetallic gray form with the direct band gap of ca. 0.1 eV constitutes an electronic ground state at T ➔ 0 K, while the metallic white tin (with closed fundamental band gap) is a high-energy polymorph, which is observed at ambient temperature conditions.

At the first sight any metal (with its band gap closed) is infinitely soft. However, albeit any metal’s softness is large as compared to those of semiconductors or insulators, it is in fact finite. Yang and Parr have derived useful equation relating hardness of a metal to the density of states, DOS, at its Fermi level, E_F_ [11]:

1 $$ \upeta =\mathrm{DOS}{\left({\mathrm{E}}_{\mathrm{F}}\right)}^{\hbox{--} 1} $$

This intuitive formula informs that a metal which has many electrons at its Fermi level (and thus ready to be used for dielectric polarization) is softer than the one, which has only small electron density available for the same reason. Majority of known “good” metals, such as Li or Cu, have very small DOS(E_F_), which stems from appreciable width of electronic bands in question. For example, valence and conduction band dispersion in metals often exceeds 5 eV and at high pressure it may surpass 10 eV. Consequently, most metals host charge carriers with small effective mass; such light electrons do not readily couple to lattice vibrations (phonons) of much heavier atomic cores and thus, according to the BCS theory of superconductivity [12], the prospect for high values of T_C_ in conventional metals is rather limited. Hence, as it has been early noticed, the “better” the metal, the “worse” is T_C_ in the superconducting state. Consequently, to this day superconductivity has not been observed for the best metallic conductors such as Cu, Au or Au, while the observation of the superconducting transition for lithium (at *T* < 0.4 mK!) has stirred the community [13].

Given the above, it may seem that the scarcity of high-temperature superconductors may be rationalized simply by the rarity of high-density of state metals in Nature – as requested by the Maximum Hardness Principle [9]. Here we computationally test this hypothesis by calculating the crystal and electronic structures as well as stability of the simplest parent compound of an oxocuprate superconductor, the layered form of CaCuO_2_ [14, 15], and its electron- and hole-doped analogues. We also study the disproportionation tendency for doped phases, as well as their electronic structures, and in particular DOS(E_F_) values.

## Methods

Density Functional Theory (DFT) calculations were performed using the plane-wave VASP code (https://www.vasp.at/) [16–19]. Both spin-polarized and nonpolarized calculations were performed for all models containing Cu(II) cations with d^9^ electronic configuration, namely for CaCuO_2_ and its 12.5% hole- and electron-doped variants. Both sets of data are presented in this work. The hole- and electron-doping was simulated by exchanging one of the Ca(II) centers in a 2x2x2 CaCuO_2_ supercell (Z = 8) by Na(I) and La(III), respectively. Two electronic solutions were calculated for doped systems: a metallic solution without any spin polarization, and a magnetic model. The magnetic model (see Fig. 2 left panel, below) consisted of 2x2x2 CaCuO_2_ supercell (Z = 8) with intralayer AFM ordering and FM coupling of the CuO_2_ layers. The same magnetic structure was assumed for the hole- and electron-doped CaCuO_2_ systems, while one of the eight Cu centers in the supercell was left non-polarized to mimic the changed electronic situation due to the hole (Cu(II) ➔ Cu(III)) and electron-doping (Cu(II) ➔ Cu(I)), respectively.

All structural models were first optimized using the PBEsol functional [20]. Then single-point total energy and electronic density of states were calculated for the optimized models using hybrid DFT functional HSE06 [21]. The thresholds for electronic and ionic convergence were set to 10^−7^ and 10^−5^ eV, respectively. In case of extremely computational resources-demanding hybrid DFT calculations the threshold for electronic convergence was reduced to 10^−5^ eV. Plane-wave cutoff energy was set to 520 eV, and k spacing to 0.2 Å^−1^. The DFT + U structure optimizations utilized U(Cu_3d_) = 9 eV, J(Cu_3d_) = 1 eV.

**Fig. 1:**
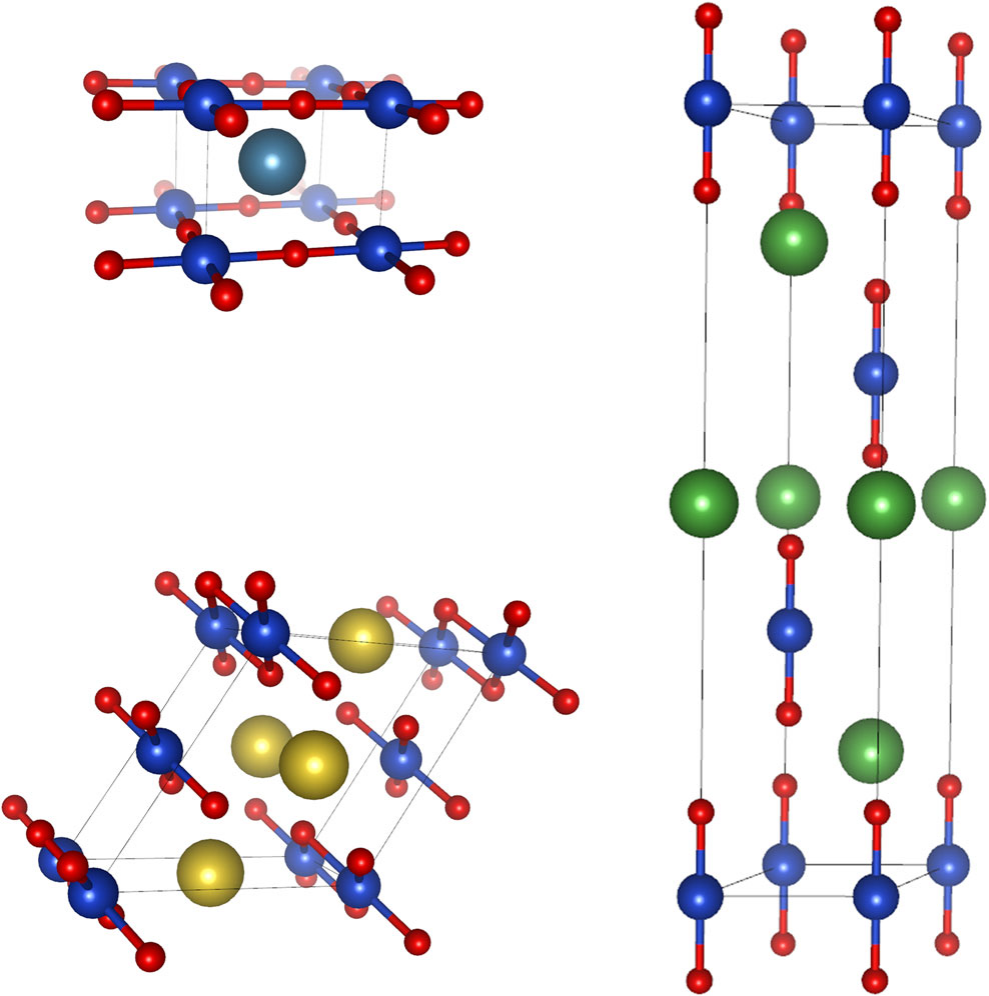
Crystal structures relevant to this work: infinite layer CaCu(II)O_2_ – top left [22], NaCu(III)O_2_ – bottom left [23], and LaCu(I)O_2_ – right [24]. The Cu–O bonds were drawn, while other bonds were omitted

The crystal structures, which subsequently underwent full structure and energy optimizations were taken from the ICSD depository: structure No.86544 for CaCu(II)O_2_ [22], 80,561 for NaCu(III)O_2_ [23] and 18,102 for LaCu(I)O_2_ [24] (Fig. 1).

## Results

### Crystal structures for reference compounds

The unit cell of CaCuO_2_ optimized in its metallic state shows the CuO bond length of 1.913 Å, thus not far from the experimental value of 1.928 Å (Table 1).

**Table 1:** Selected optimized and experimental (bracketed [22–24]) structural parameters of antiferromagnetic CaCu(II)O_2_, as well as diamagnetic NaCu(III)O_2_ and LaCu(I)O_2_

Parameter	CaCu(II)O_2_	NaCu(III)O_2_	LaCu(I)O_2_
a /Å	3.8255 (3.8555)	6.4614 (6.363)	3.8189 (3.83)
b /Å	3.8255 (3.8555)	2.7457 (2.753)	3.8189 (3.83)
c /Å	3.1517 (3.1798)	6.1846 (6.110)	16.8090 (17.10)
β /deg	90 (90)	122.632 (120.78)	90 (90)
V /Å^3^	46.125 (47.27)	92.402 (91.95)	212.300 (217.23)
R(Cu–O) /Å	1.913 (1.928)	1.834 (1.846)	1.795 (1.847)

The optimized unit cell vectors of NaCu(III)O_2_ and LaCu(I)O_2_ also fall rather close to the experimental values; and their discrepancies partially cancel out as far as volume and relevant CuO bond lengths are considered.

### Crystal structures of 1/8-doped compounds

Doping to [CuO_2_] sheets in cuprates may be realized via several different approaches, such as introducing additional O or F atoms to the structure (for hole doping), applying external pressure (to inject charge from charge reservoir layers), or performing isoelectronic substitution, e.g. La(III) ➔ Ba(II), La(III) ➔ Sr(II), Ca(II) ➔ Na(I), Nd(III) ➔ Ce(IV), etc. The last method is very elegant and it works well for many other families of superconductors, such as bismuthates (Ba(II) ➔ K(I)), plumbates, etc. This type of doping may also rather easily be reproduced using theoretical calculations for periodic systems (while assuming crystallinity i.e. ordering at the dopant sites), and we have chosen to mimic this particular method in our computational modelling approach.

Correspondingly, in a subsequent step, we have mimicked the formation of the high-T_C_ superconductor by substituting 1/8 of Ca atoms in the CaCuO_2_ structure by either Na (this corresponds to hole doping as 1/8 Cu(II) formally becomes Cu(III)) or La (this stands for electron doping) [25]. The choice of a doping level set at 1/8 comes from purely technical reasons as such doping may easily be modelled in theory by using of the 2x2x2 supercell of CaCuO_2_ (Z = 8) while still keeping symmetry high [26–31]. Note that superconductivity in oxocuprate materials arises for a quite broad doping level (“superconducting domes”), with T_C_ maximized at doping level close to 15–16% i.e. ±1/6 (which, simply, is less convenient to model than 12.5% adopted here). Thus, the doped structures were calculated here using periodic boundary conditions and assuming ordering of dopant atoms on a primitive tetragonal lattice within the 2x2x2 supercell of CaCuO_2_. Due to symmetry constraints this model does not permit a genuine localization of Cu(I) or Cu(III) sites, which would obviously be an undesired feature of the model.

At the doping level as small as 3% (for hole-doped materials) and around 10% (for electron-doped systems) the long-range magnetic ordering disappears and superconductivity arises, while the electronic state above the T_C_ value corresponds to a metal. Therefore, we have carried out calculations for the 12.5%-doped phases.

**Fig. 2:**
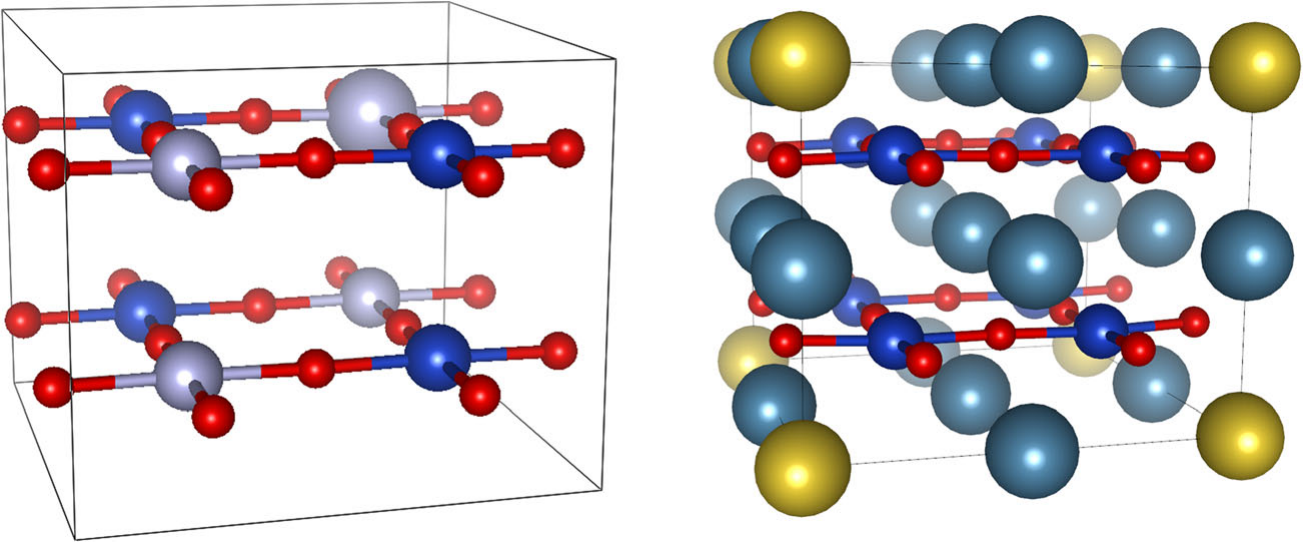
(left) Magnetic model used in spin-polarized calculations of CaCuO_2_ and its doped variants. The small red balls are oxygen atoms, and light and dark blue balls represent Cu atoms with spin up and down, respectively (he biggest light-blue ball represents Cu center, on which a hole (or an extra electron) was imposed. The cationic layers (containing Ca, La and Na) are left out for clarity). (right) Optimized crystal structure of 1/8-doped CaCu(II)O_2_: dopant atoms sit in (0,0,0) and to allow for this, the original CaCuO_2_ cell contents is shifted by the (0.5,0.5,0.5) vector

The crystal structure model of 1/8-doped CaCu(II)O_2_ is showed in Fig. 2 (right), and the respective optimized parameters are listed in Table 2.

**Table 2:** The optimized selected structural parameters of 1/8-doped CaCu(II)O_2_ (Na doping & La doping) as compared to the undoped system; the lattice vectors of doped systems were divided by 2 for comparison; the CuO bond lengths for doped systems were additionally averaged for each Cu site to mimic the “perfectly disordered” model

Parameter	CaCu(II)O_2_	(NaCuO_2_)_1/8_(CaCuO_2_)_7/8_		(LaCuO_2_)_1/8_(CaCuO_2_)_7/8_	
		Without spin polarization	Magnetic model	Without spin polarization	Magnetic model
a /Å	3.826	3.804	3.786	3.855	3.832
b /Å	3.826	3.804	3.786	3.855	3.832
c /Å	3.152	3.173	3.178	3.171	3.169
V /Å^3^	46.125	45.896	45.553	47.128	46.536
R(Cu–O) /Å	1.913	1.890, 1.917	1.879–1.910	1.913, 1.943	1.848–1.992
R_aver_(Cu–O) /Å	1.913	1.903	1.894	1.928	1.916

As expected, the hole- or electron-doping to CaCuO_2_ by substituting Ca(II) with nearly identically sized Na(I) or La(III) cations, respectively [25], does not yield large structural changes to the rigid structures featuring a network of rather covalent CuO bonds. However, although the changes are small, yet they may be rationalized: introduction of holes to the [CuO_2_] sheets (i.e. a partial depopulation of the Cu-O σ* states) leads to a decrease of tetragonal lattice parameters by some 0.02–0.04 Å thus to a simultaneous shorting of intra-sheet CuO bond lengths by ca. 0.01–0.02 Å. On the other hand, introduction of electrons to the sheets is reflected in elongation of the tetragonal lattice parameter by up to ca. 0.03 Å, which translates to elongation of the CuO bonds (on average) by up to half of this value. These changes nicely agree with what is expected from molecular orbital theory considerations, as bond elongation is expected upon gradual population of the Cu-O σ* states. Additionally, the small values of changes reflect the well-known rigidity of the [CuO_2_] manifold.

In both cases (electron and hole doping) the value of unit cell vector ***c*** slightly increases; this is supposedly due to a slightly larger size of the dopant cations as compared to pristine Ca(II); ***c*** increases so that the strain may be relaxed. However, the net effect of all these changes on volume of the unit cell is relatively small: −0.5% for Na-doping and + 2.2% for La-doping.

### Energetics of the formation reaction

Of immediate interest is energetics for the reaction:

2 $$ 1/8\ {\mathrm{MCuO}}_2+7/8\ {\mathrm{CaCuO}}_2\rightarrow {\left({\mathrm{MCuO}}_2\right)}_{1/8}{\left({\mathrm{CaCuO}}_2\right)}_{7/8}. $$

Table 3 lists the calculated energies of the substrates and products of this reaction separately for M = Na and M = La, without zero-point vibrational energy correction (which is usually very small except for hydrides), as well as reaction energy (Eq. 2).

**Table 3:** The calculated energy of the substrates and products of Eq. 2 [eV], as well as energy of reaction (Eq. 2)

M	1/8 MCuO_2_ + 7/8 CaCuO_2_	(MCuO_2_)_1/8_(CaCuO_2_)_7/8_		Δ_prod–subst_ / eV	
		Without spin polarization	Magnetic model	Without spin polarization	Magnetic model
Na	−27.560	−27.184	−27.418	+0.376	+0.141
La	−28.790	−28.386	−28.642	+0.404	+0.149

It is clear from Table 3 that the formation of metallic (as follows from spin-unpolarized calculations) (MCuO_2_)_1/8_(CaCuO_2_)_7/8_ from 1/8 MCuO_2_ and 7/8 CaCuO_2_ precursors is energetically uphill by quite a substantial energy. For M = Na this energy is nearly identical to the energy difference between the metallic and antiferromagnetic form of CaCuO_2_, calculated here to be −0.382 eV. For M = La the energy of reaction is even larger than that, by ca. 0.02 eV. Although our initial model of the doped phases assumed their metallic character (with no magnetic interactions whatsoever), it is clear that even if short-range antiferromagnetic interactions are still present in the “weird metal” (electronic form of a superconductor for T > T_C_) yet the energy gain associated with these interactions may only constitute *a fraction* of the −0.382 eV stabilization energy; *ergo*, the doped metallic phases are *not* stable in terms of energy with respect to precursors at the left hand side of reaction equation, Eq. 2. Indeed, this is confirmed by our calculations involving spin polarization, which yield (MCuO_2_)_1/8_(CaCuO_2_)_7/8_ energetically uphill with respect 1/8 MCuO_2_ and 7/8 CaCuO_2_ by 0.14–0.15 eV; this means that about 2/3 of magnetic interactions are preserved in the doped systems, which, however, is insufficient to energetically stabilize the (MCuO_2_)_1/8_(CaCuO_2_)_7/8_ product.

This conclusion obviously holds for T ➔ 0 K and p ➔ 0 atm, i.e. ground state conditions, but it translates also to free energy due to vanishing of the pV and ST factors. Moreover, since the energy stabilization of the superconducting state with respect to a parent metal corresponds to absorption in microwave region, and thus it is of the order of 1 meV, it is clear that the doped systems in their ground superconducting state are *not* energetically and thermodynamically stable with respect to 1/8 MCuO_2_ and 7/8 CaCuO_2_ precursors, as well.

The situation would be quite different *if* CaCuO_2_ was metallic; however, it is rather an antiferromagnetic insulator and the fact that injection of holes or electrons into the [CuO_2_] sheets disrupts the perfect 2D network of antiferromagnetic interactions is the key reason why *the doped phases are metastable*.

### Electronic DOS(E_F_)

The HSE06 hybrid-functional DFT calculations allowed us to assess the size of the fundamental bandgap of the systems studied, as well as the density of states of all systems (Table 4).

**Table 4:** The calculated fundamental band gap, Δ_g_ [eV], as well as density of states at the thermodynamic Fermi level, DOS(E_F_), for the substrates and products of Eq. 2 [states eV^−1^], always for Z = 1 (per one Cu atom)

Compound	NaCuO_2_	LaCuO_2_	CaCuO_2_	(NaCuO_2_)_1/8_(CaCuO_2_)_7/8_		(LaCuO_2_)_1/8_(CaCuO_2_)_7/8_	
				Without spin polarization	Magnetic model	Without spin polarization	Magnetic model
Δ_g_	2.43	4.08	2.16	0	0	0	0.3
DOS(E_F_)	0	0	0	0.41	0.87	0.830	0*

*The DOS(E_F_) values strongly depend on the fine details of parameters used to describe band occupation and convergence algorithms; the system is on the verge of a metal [32] and being a narrow band gap semiconductor

All precursors of the reaction described by Eq. 2 are insulators, with sizeable band gaps; the calculated band gap approaches 2.2 eV for antiferromagnetic CaCuO_2_, exceeds 2.4 eV for low-spin [33] NaCuO_2_ with its totally empty d(x^2^–y^2^) / p_x_,p_y_ σ* conduction band, and is even larger than 4.0 eV for LaCuO_2_, the d^10^ system. On the other hand, metallic spin-unpolarized solutions of the doped systems show appreciable DOS(E_F_) values [34], which stems mostly from partial occupation of the nearly half-filled d(x^2^–y^2^) / p_x_,p_y_ σ* band, doped at ±12.5%. When spin polarization of the doped structures is taken into account, the Na-doped compound shows even higher (two-fold larger) DOS(E_F_) value than in the spin-unpolarized solution. On the other hand, the spin-polarized La-doped system is on the verge between the metallic and semiconducting behaviour, the DOS(E_F_) values strongly depending on the fine details of parameters used to describe band occupation and convergence algorithms.

### MHP considerations

Analysis of the doped systems using ramifications of the MHP is pretty straightforward, regardless of the nature of doping (holes, electrons): the chemical reaction proceeding according to Eq. 2 is energetically uphill (section “Methods”) since it corresponds to a formation of electronically soft *metals* or *narrow-gap semiconductors* (though the metallic behaviour is more likely for La-doped system [32]) starting from much harder *insulators*. Progress of reaction (Eq. 2) is obviously forbidden by the MHP. In fact, the opposite reaction should occur, i.e. a once-prepared doped system should exhibit tendency towards phase separation, in which all doping should be eliminated, and all extra charge (electrons or holes) should be localized within a separate minority phase (LaCuO_2_ or NaCuO_2_, respectively). In other words, a doped system would prefer to disproportionate and place two different valences of copper in two distinct crystalline phases.

But, this leads us to an important question, how a *metastable superconductor* may be manufactured at all?

The success of preparation of superconductor precursor, i.e. a metal (or a heavily doped semiconductor with many charge carriers available for pairing) seems to originate from using of (p,T) conditions which are very far from p➔ 0 atm, and T➔ 0 K. A typical method of manufacturing relies on multiple firing up of a mixture of oxides, and once the correct crystal structure has been reached, oxygen gas is used at high temperature to dope the system with holes. Once the extra O atoms are trapped in the structure (usually in the reservoir layers), the sample is cooled down, and the escape of O atoms become impossible even if thermodynamics dictates so. Moreover, the reconstruction of the entire crystal lattice (which is needed for the reaction reversed to that described by Eq. 2) to occur, is also prevented by huge barriers associated with ionic mobility within a rigid lattice of the ceramic oxide. Yet another method of preparation utilizes F_2_ gas as a dopant, and – due to high reactivity of this element – the doping reaction is usually downhill in energy; again, the main framework of the copper oxide is preserved, and energy barriers for phase separation prevent formation of insulating metal fluorides (precisely this would be expected based on chemical intuition, and MHP considerations as well).

## Conclusions and prospect

Summarizing, oxocuprate superconductors are *not* stable at p ➔ 0 atm and T ➔ 0 K conditions. Use of non-equilibrium conditions for their preparation is the key to achieving the “weird metal” precursor which may enter the superconducting state upon cooling below T_C_. Since the methods for preparation of iron pnictides and chalcogenide superconductors do not differ much from those used for oxocuprates, and the undoped compounds show strong 2D antiferromagnetic interactions (just like CaCuO_2_) it is assumed without a proof that the situation for iron superconductors is similar. Moreover, the superconducting H_3_S [4] owes its stability only to ultra-high pressure conditions (*p* > 2 mln atm), and it readily decomposes to H_2_S and ½ H_2_ (both systems are broad band gap insulators) when external constrains are released. Supposedly, the key to all high-T_C_ superconductors sits in their metastable character, and hindering of the phase separation either due to rigidity of crystal lattice alone, or by using of an external pressure. Still, many of these systems are known to show propensity towards phase micro-separation or superstructure formation, which are the ways the system tries to minimize energy, and obey MHP to some extent. Similar problems may be anticipated for other exotic systems, where superconductivity is searched, as well [35].

Nevertheless, since the key preoccupation of chemists is the successful preparation of novel compounds, *majority of which are metastable*, hence, justifiably, hope for room-T_C_ superconductivity never dies.
